# The role of striated muscle Pik3r1 in glucose and protein metabolism following chronic glucocorticoid exposure

**DOI:** 10.1016/j.jbc.2021.100395

**Published:** 2021-02-07

**Authors:** Tzu-Chieh Chen, Taiyi Kuo, Mohamad Dandan, Rebecca A. Lee, Maggie Chang, Sneha D. Villivalam, Szu-Chi Liao, Damian Costello, Mahalakshmi Shankaran, Hussein Mohammed, Sona Kang, Marc K. Hellerstein, Jen-Chywan Wang

**Affiliations:** 1 Metabolic Biology Graduate Program, University of California Berkeley, Berkeley, California, USA; 3 Endocrinology Graduate Program, University of California Berkeley, Berkeley, California, USA; 2 Department of Nutritional Sciences & Toxicology, University of California Berkeley, Berkeley, California, USA

**Keywords:** glucocorticoids, glucocorticoid receptor, Pik3r1, insulin resistance, protein synthesis, striated muscle, skeletal muscle, AcH3, acetylated histone H3, AcH4, acetylated histone H4, ChIP, chromatin immunoprecipitation, DEX, dexamethasone, GA, gastrocnemius, GR, glucocorticoid receptor, GRE, glucocorticoid response element, pAkt, phosphorylated Akt, PI3K, phosphoinositide 3-kinase, *Pik3r1*, phosphoinositide-3-kinase regulatory subunit 1, PTEN, phosphatase and tensin homolog

## Abstract

Chronic glucocorticoid exposure causes insulin resistance and muscle atrophy in skeletal muscle. We previously identified phosphoinositide-3-kinase regulatory subunit *1* (*Pik3r1*) as a primary target gene of skeletal muscle glucocorticoid receptors involved in the glucocorticoid-mediated suppression of insulin action. However, the *in vivo* functions of Pik3r1 remain unclear. Here, we generated striated muscle-specific *Pik3r1* knockout (MKO) mice and treated them with a dexamethasone (DEX), a synthetic glucocorticoid. Treating wildtype (WT) mice with DEX attenuated insulin activated Akt activity in liver, epididymal white adipose tissue, and gastrocnemius (GA) muscle. This DEX effect was diminished in GA muscle of MKO mice, therefore, resulting in improved glucose and insulin tolerance in DEX-treated MKO mice. Stable isotope labeling techniques revealed that in WT mice, DEX treatment decreased protein fractional synthesis rates in GA muscle. Furthermore, histology showed that in WT mice, DEX treatment reduced GA myotube diameters. In MKO mice, myotube diameters were smaller than in WT mice, and there were more fast oxidative fibers. Importantly, DEX failed to further reduce myotube diameters. *Pik3r1* knockout also decreased basal protein synthesis rate (likely caused by lower 4E-BP1 phosphorylation at Thr37/Thr46) and curbed the ability of DEX to attenuate protein synthesis rate. Finally, the ability of DEX to inhibit eIF2α phosphorylation and insulin-induced 4E-BP1 phosphorylation was reduced in MKO mice. Taken together, these results demonstrate the role of Pik3r1 in glucocorticoid-mediated effects on glucose and protein metabolism in skeletal muscle.

Glucocorticoids are steroid hormones that play important roles in regulating whole body metabolism under stress conditions, mainly by mobilizing energy sources to face severe challenges. In skeletal muscle, glucocorticoids inhibit protein synthesis, facilitate protein degradation, and suppress glucose utilization. Amino acids generated from glucocorticoid-induced protein mobilization are the precursors for hepatic gluconeogenesis and inhibiting glucose utilization raises plasma glucose concentrations (1, 2). While these glucocorticoid effects are critical for metabolic adaptations during stress, chronic/excess glucocorticoid exposure causes hyperglycemia, insulin resistance, and muscle atrophy (1, 3, 4, 5).

Glucocorticoids convey their functions through the glucocorticoid receptor (GR), which is a transcription factor that binds to genomic glucocorticoid response elements to modulate the transcriptional rate of its target genes. Thus, GR primary target genes initiate the physiological actions of glucocorticoids. To understand the mechanisms underlying glucocorticoid actions in skeletal muscle, we previously used a combination of global gene expression analysis and chromatin immunoprecipitation sequencing to identify a list of potential GR primary target genes in murine C2C12 myotubes (6). *Phosphoinositide-3-kinase regulatory subunit 1* (*Pik3r1*, a.k.a. *p85α*) is one of these potential GR primary target genes (6) and encodes a regulatory subunit of phosphoinositide 3-kinase (PI3K), which is composed of a regulatory subunit (Pik3r1, Pik3r2, or Pik3r3) and a catalytic subunit (Pik3ca1 or Pik3ca2) (7, 8). When insulin signaling is activated, PI3K is recruited to the activated insulin receptor substrate 1 to convert phosphatidylinositol-4, 5 bisphosphate to phosphatidylinositol-3, 4, 5 triphosphate. Binding to phosphatidylinositol-3, 4, 5 triphosphate at the plasma membrane is required to activate protein kinase Akt (9), a key signaling molecule in mediating the metabolic functions of insulin. Though Pik3r1 is a key component of the insulin pathway, overexpression of monomeric Pik3r1 was found to suppress insulin signaling in myotubes and hepatocytes (10, 11). Conversely, Pik3r1 deficiency has been shown to improve insulin sensitivity (12, 13). Several mechanisms have been proposed for the inhibitory effect of excess Pik3r1 on insulin signaling. First, monomeric Pik3r1 competes with heterodimeric PI3K for binding to insulin receptor substrate-1 to suppress insulin signaling (14). Alternatively, Pik3r1 could enhance the activity of phosphatase and tensin homolog (PTEN) to inhibit PI3K (15). Another report shows that homodimeric but not monomeric Pik3r1 suppresses PI3K by protecting PTEN from ubiquitin-mediated proteasomal degradation. Further, the p85alpha homodimer enhances lipid phosphatase activity and membrane association of PTEN (16).

We previously found that the overexpression of Pik3r1 in C2C12 myotubes reduced cell diameter while reduction in Pik3r1 expression compromised glucocorticoid suppression of insulin signaling (6). To further investigate the role of Pik3r1 in glucocorticoid actions in skeletal muscle, we created striated muscle–specific *Pik3r1* knockout mice (MKO) (17). We treated MKO and *Pik3r1*^*flox/flox*^ mice (will be referred as wildtype, WT, mice in this report) with or without a synthetic glucocorticoid, dexamethasone (DEX), and studied the effects on insulin signaling in metabolic tissues, including liver, gastrocnemius (GA) muscle, and epididymal white adipose tissue. We also examined the effect of striated muscle Pik3r1 deletion on systemic glucose and insulin tolerance. In addition, using stable isotope labeling techniques and tandem mass spectrometry, we analyzed the DEX effects on protein synthesis rates in GA muscle of WT and MKO mice. Finally, we investigated the signaling pathways involved in the regulation of protein synthesis and conducted histological analysis of the GA muscle in WT and MKO mice.

## Results

### GR increased Pik3r1 gene transcription and protein expression in mouse gastrocnemius muscle

**Figure 1:**
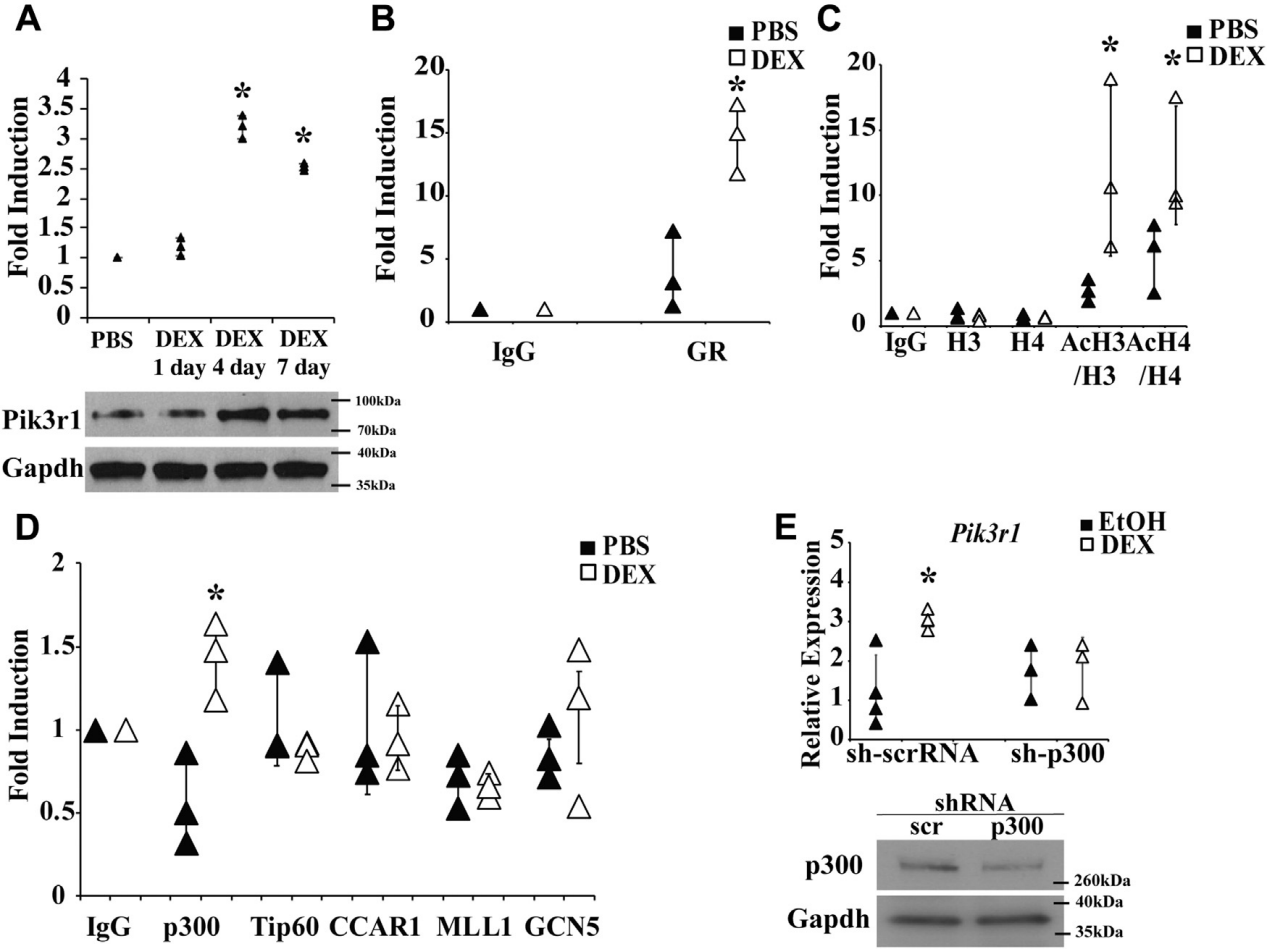
**DEX induces Pik3r1 expression in skeletal muscle *in vivo* and P300 mediates the transactivation of Pik3r1 in skeletal muscle male 8-week-old WT mice were treated with PBS or 5 mg/kg of daily dexamethasone (DEX) through IP injection for 1, 4, 7 days**. The Pik3r1 expression was examined by immunoblot in (*A*) gastrocnemius muscle and normalized to internal control Gapdh. Representative immunoblots are shown (n = 3). Error bars represent the SD of relative Pik3r1 expression level (DEX *versus* PBS), and ∗*p* < 0.05 male 8-week-old Pik3r1 Flox (WT) mice were treated with 5 mg/kg of DEX for 4 days. Then, their GA muscles were collected. ChIP experiments were performed on these GA muscles to study the recruitment of glucocorticoid receptor (GR) (*B*), the histone modification (*C*), and the recruitment of transcription cofactor p300, Tip60, CCAR1, MLL1, and CGN5 (*D*) on glucocorticoid response element of Pik3r1. Primer flanking the Pik3r1 glucocorticoid response element and Rpl19 (internal control) were used in qPCR. Error bars represent the SD of relative fold enrichment compared to IgG control from three independent experiments and ∗*p* < 0.05. *E*, C2C12 myoblasts were infected with lentivirus particles expressing scramble sh-RNA (sh-scrRNA, control) or sh-p300. After puromycin selection, cells were differentiated into myotubes and were then treated with 1 μM DEX or EtOH for 6 h. QPCR was performed to monitor the expression of *Pik3r1* and Western blot was shown to confirm the knockdown of p300. Error bars represent the SD of fold induction of *Pik3r1*. ∗*p* < 0.05.

We previously showed that *Pik3r1* gene expression was elevated in mouse GA muscle upon DEX treatment (6). We examined whether Pik3r1 protein expression was indeed increased by DEX treatment. Male WT mice were injected intraperitoneally with DEX or PBS daily for 1, 4, or 7 days. GA muscles were collected to detect the expression of Pik3r1 using immunoblotting. We found that Pik3r1 expression was significantly increased upon DEX treatment for 4 and 7 days (Fig. 1*A*).

To confirm the activation of *Pik3r1* gene transcription by GR *in vivo*, chromatin immunoprecipitation (ChIP) was performed to test the recruitment of GR to the glucocorticoid response element (GRE) of *Pik3r1* gene in mouse GA muscle. Eight-week-old male WT mice were injected intraperitoneally with PBS or DEX for 4 days before tissue collection for ChIP given that Pik3r1 had the highest protein expression with 4 days of DEX treatment. The glucocorticoid response element of *Pik3r1* has been located between −43,938 and −43,924 upstream of the mouse *Pik3r1* gene (6). We found that GR was significantly recruited to the *Pik3r1* GRE by DEX treatment (approximately 15-fold comparing to IgG ChIP control) (Fig. 1*B*). Interestingly, GR was also recruited to the *Pik3r1* GRE in PBS-treated animals to a lesser degree (approximately 5-fold, Fig. 1*B*). This suggests that plasma corticosterone levels are enough to activate *Pik3r1* gene transcription through GR.

Transcriptional activation is associated with elevated histone acetylation in the enhancer regions (18, 19). We performed ChIP to monitor the acetylated histone H3 (AcH3) and H4 (AcH4) and total H3 and H4 at the *Pik3r1* GRE in the GA muscle of PBS and DEX-treated WT mice. The ratios of AcH3/H3 and AcH4/H4 represent the degrees of the histone acetylation in H3 and H4, respectively. As shown in Figure 1*C*, the level of total histone H3 and H4 was not significantly affected by DEX treatment. However, comparing to IgG control, the ratios of AcH3/H3 and AcH4/H4 were significantly increased by 8 and 10 folds with DEX treatment, respectively (Fig. 1*C*). In PBS treated animals, the ratios of AcH3/H3 and AcH4/H4 were also significantly higher than the IgG ChIP control (approximately 3 and 7 folds, respectively, Fig. 1*C*). This observation was consistent with the finding that GR was also recruited to the *Pik3r1* GRE under PBS treatment. Nonetheless, the ratios of AcH3/H3 and AcH4/H4 were higher in DEX-treated mice than those of PBS-treated mice. This was in agreement with a stronger GR recruitment to the *Pik3r1* GRE under DEX treatment (Fig. 1*C*). We next examined which histone acetyltransferase is recruited to the *Pik3r1* GRE using ChIP. We found that p300 but not Tip60 and GCN5 was significantly recruited to the GRE upon DEX treatment (Fig. 1*D*). These results suggested that p300 accounted for the higher histone acetylation status at the GRE upon DEX treatment. Notably, neither p300, Tip60, nor GCN5 were recruited to the GRE upon PBS treatment (Fig. 1*D*). These results suggest that histone acetyltransferase(s) other than these three are involved in the acetylation of the *Pik3r1* GRE in PBS-treated mice.

To test whether p300 is involved in GR-activated *Pik3r1* gene transcription, C2C12 myoblasts were infected with lentivirus expressing scramble small hairpin RNA (sh-scrRNA, control) or shRNA against p300 (sh-p300). After puromycin selection, cells were differentiated into myotubes, then treated with DEX or EtOH for 6 h. RNA was isolated from these cells, and qPCR was performed to monitor the expression of *Pik3r1*. In sh-scrRNA expressing C2C12 myotubes, DEX treatment increased the expression of Pik3r1 approximately 3-fold (Fig. 1*E*). However, in sh-p300 expressing C2C12 myotubes, such DEX effect was abolished (Fig. 1*E*). The Western blot showed that p300 was efficiently reduced by RNAi (Fig. 1*E*).

### The ability of DEX to suppress insulin signaling was attenuated in gastrocnemius muscle of MKO mice

**Figure 2:**
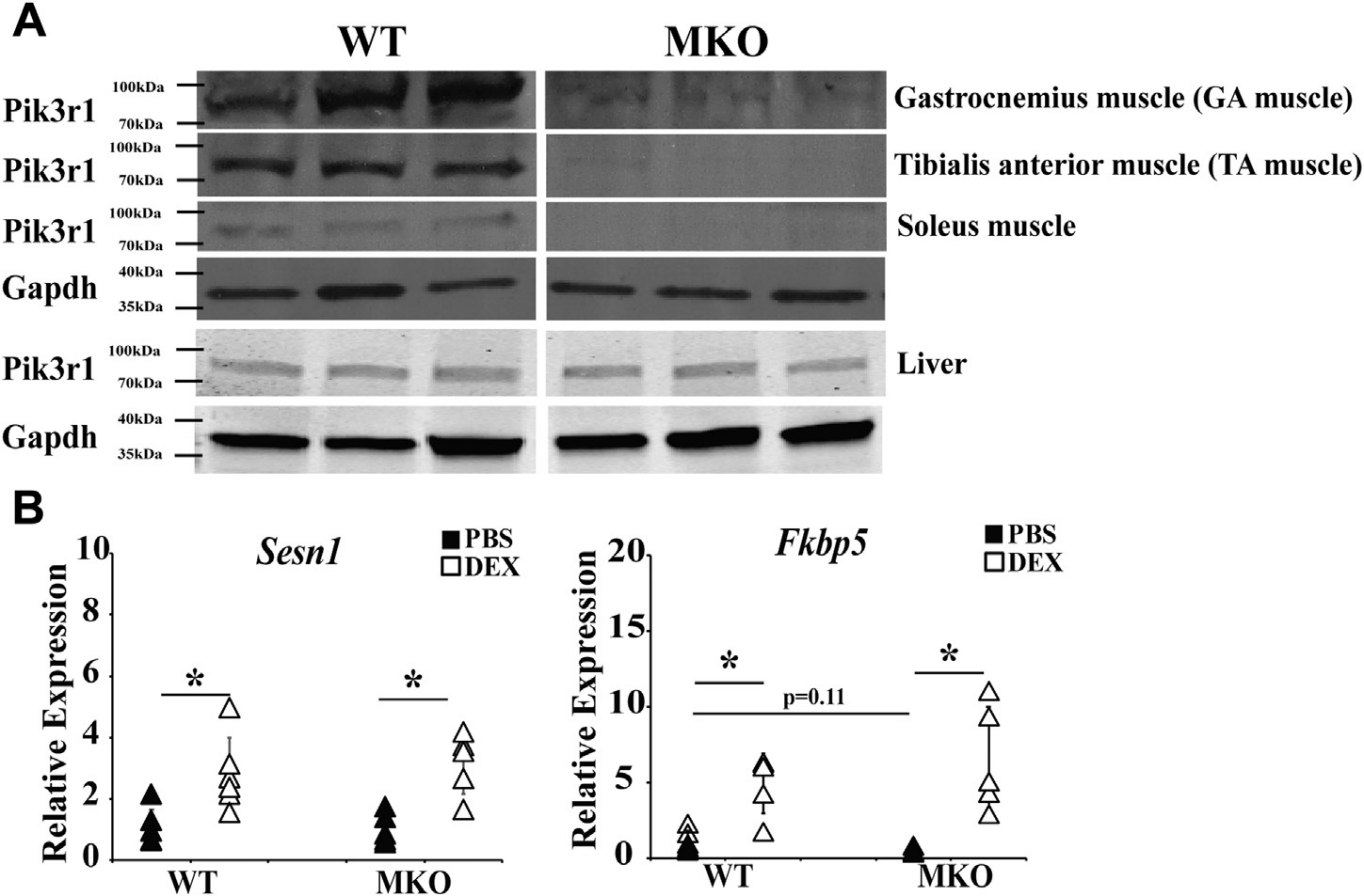
**GC-induced gene expression in MKO mice**. Muscle specific Pik3r1 knockout (MKO) mice were generated. *A*, the expression of Pik3r1 in gastrocnemius (GA) muscle, tibialis anterior (TA) muscle, and soleus muscle of WT and MKO was examined by immunoblots and normalized to internal control Gapdh. Representative immunoblots are shown (n = 3). *B*, the expression of Sesn1 and Fkbp5 was evaluated in WT and MKO mouse gastrocnemius muscle treated with PBS or DEX (10 mg/kg bodyweight) in drinking water for 1 week. Error bars represent SD, n = 5 to 6 and ∗*p* ≤ 0.05. DEX, dexamethasone.

We generated striated muscle–specific *Pik3r1* knockout mice (MKO) by crossing *Pik3r1*^*flox/flox*^ mice with transgenic mice carrying muscle creatine kinase promoter driving the expression of Cre recombinase (20). In MKO mice, Pik3r1 expression was indeed depleted in skeletal muscles, including GA muscle, tibialis anterior muscle, and soleus muscle (Fig. 2*A*). Notably, Pik3r1 expression was similar in the liver of WT and MKO mice (Fig. 2*A*). These results validated the specific deletion of *Pik3r1* in the striated muscle. We also tested the ability of DEX to induce the expression of GR primary target gene in GA muscle of WT and MKO mice. WT and MKO mice were treated with PBS or DEX for 7 days. GA muscle RNA was isolated, and qPCR was performed to examine the DEX induction of two previously identified GR primary target genes, *Fkbp5* (21) and *Sesn1* (6). DEX treatment increased approximately 3-fold of expression of *Sesn1* in WT mice. In MKO mice, similar DEX response was observed (Fig. 2*B*). DEX treatment elevated the expression of *Fkbp5* approximately 5-fold in WT mice (Fig. 2*B*). The basal *Fkbp5* expression was lower in MKO mice, though not statistically significant (*p* = 0.11). DEX treatment also efficiently increased *Fkbp5* expression in MKO mice (Fig. 2*B*). These results indicated that depleting Pik3r1 expression did not affect general GR activity in GA muscle.

**Figure 3:**
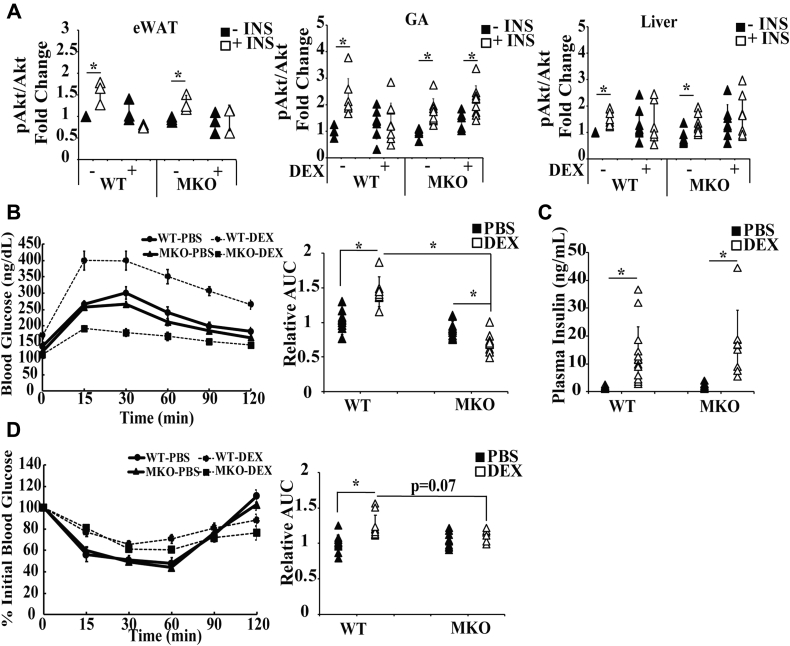
**GC-induced glucose intolerance is compromised in MKO mice.***A*, male 8-week-old WT and MKO mice were treated with 10 mg/kg of PBS or DEX in drinking water for 1 week. On the last day, mice were injected intraperitoneally with insulin (1 unit/body weight) for 10 min, and then various tissues were collected. ELISA kits were used to monitor the level of Akt and phosphor-Akt in eWAT, liver, and GA muscle. The results are presented as relative pAkt/AKt level. Error bars represent the SD, n = 3 to 9 and ∗*p* ≤ 0.05 comparing no insulin treatment to insulin treatment. *B*, male 8-week-old WT mice and MKO mice were treated with 10 mg/kg of DEX for 7 days. On the last day, mice were fasted for 15 h, and the IPGTT was performed. Relative area under curve (AUC) for IPGTT results (relative to PBS-treated WT mice). Error bars represent the SD, n = 6–12 and ∗*p* ≤ 0.05. *C*, plasma insulin level was measured before glucose injection (0 min time point). Error bars represent the SD, n = 6 to 12 and ∗*p* ≤ 0.05. *D*, ITT was performed in mice as described in Methods. ITT results were depicted as percentage of initial plasma glucose level (the plasma glucose level before insulin injection). Error bars represent the SD, n = 3 to 7. Relative area under curve (AUC) for ITT results (relative to PBS-treated WT mice) is shown. Error bars represent the SD, n = 6 to 11 and ∗*p* ≤ 0.05. DEX, dexamethasone; IPGTT, intraperitoneal glucose tolerance test; ITT, insulin tolerance test.

WT and MKO mice treated with or without DEX for 7 days were injected with insulin for 10 min. GA muscle, liver, and epididymal white adipose tissue were isolated, and the activity of a key molecule in insulin signaling, Akt, was monitored. For Akt activity, we monitored the levels of phosphorylated Akt (pAkt) at serine 473 residue (22) and total Akt. The activity of Akt was represented by the ratio of pAkt and Akt. Insulin treatment elevated pAkt/Akt ratio in GA muscle, liver, and epididymal white adipose tissue of WT mice (Fig. 2*B*). In MKO mice, insulin treatment significantly increased pAkt/Akt ratio in GA muscle, liver, and epididymal white adipose tissue (Fig. 3*A*). We found that DEX treatment reduced Akt activity in all three tissues of WT mice (Fig. 3*A*). In epididymal white adipose tissue and liver of MKO mice, DEX treatment was still able to inhibit Akt activity (Fig. 3*A*). However, in GA muscle, the ability of DEX to reduce insulin stimulated Akt activity was significantly attenuated (Fig. 3*A*). These results demonstrate that Pik3r1 deficiency in striated muscle reduced the DEX effect on insulin action in GA muscle but not in other insulin responsive metabolic tissues, such as epididymal white adipose tissue and liver.

### DEX-induced glucose and insulin intolerance were improved in MKO mice

To examine whether Pik3r1 deletion in striated muscle affects the ability of DEX to modulate insulin sensitivity, WT and MKO mice were treated with or without DEX for 1 week. After 15 h fasting, intraperitoneal glucose tolerance test was performed in these mice. In WT mice, DEX treatment caused glucose intolerance and hyperinsulinemia. (Fig. 3, *B* and *C*). In contrast, in MKO mice, although DEX treatment still caused hyperinsulinemia (Fig. 2*D*), DEX-induced glucose tolerance was significantly improved (Fig. 2*D*). We also performed insulin tolerance test in WT and MKO mice treated with or without DEX for 1 week. We found that DEX treatment resulted in insulin intolerance in WT mice (Fig. 3*D*). In MKO mice, DEX treatment still caused insulin intolerance, but to with a lesser degree (Fig. 3*D*). Thus, DEX effect on insulin tolerance was somewhat reduced in MKO mice (Fig. 3*D*). Without DEX treatment, MKO and WT mice had similar glucose and insulin tolerance and plasma insulin levels were similar (Fig. 3, *B*–*D*). These results are in agreement with the previous report (17). Overall, our results demonstrate that Pik3r1 depletion in striated muscle attenuates the DEX treatment-induced glucose and insulin intolerance.

### Proteome dynamics of WT and MKO mice gastrocnemius muscle treated with DEX

**Figure 4:**
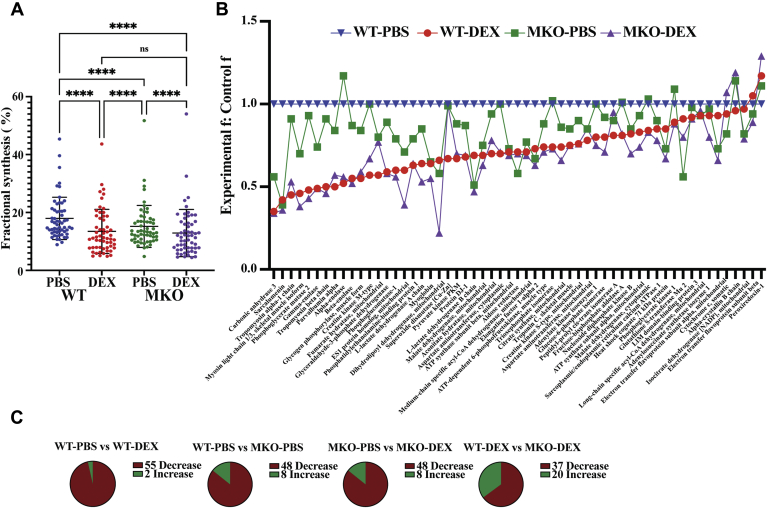
**Proteome dynamics of WT and MKO mice gastrocnemius muscle treated with DEX. male 8-week-old WT and MKO mice were treated with 10 mg/kg of PBS or DEX in drinking water for 10 days**. Heavy water labeling was conducted in final 7 days. To determine the difference in protein fractional synthesis rates (f), four groups were categorized as the following: wildtype controls treated with PBS (WT-PBS, n = 6), wildtype treated with DEX (WT-DEX, n = 6), MKO treated with PBS (MKO-PBS, n = 5), and MKO treated with DEX (MKO-DEX, n = 5). *A*, average gastrocnemius protein fraction synthesis in WT and MKO animals treated with DEX (n ≥ 3). *Bar graph* displays the mean fractional synthesis ±SD for 57 proteins common among each group. The average fractional synthesis values were 18.0%, 13.5%, 15.2%, and 12.9% for WT-PBS, WT-DEX, MKO-PBS, and MKO-DEX treated mice, respectively. The difference in the average protein fractional synthesis were assessed by ANOVA followed by Benjamini and Hochberg test for multiple comparisons (FDR = 0.05) using Prism V8.0. The percent change of the DEX effect on lowering gastrocnemius muscle protein synthesis in WT-PBS *versus* WT-DEX was −28.1% but was reduced to −17.9% in the MKO-PBS *versus* MKO-DEX. This effect remained significant (*p* < 0.04, Student *t* test with Welch’s correction). The values for MKO-PBS were significantly lower than for WT-PBS (15.5% reduced [15.2%/18.0%], *p* ≤ 0.0001). ∗∗∗∗*p* ≤ 0.0001. *B*, relative comparison in the degree change of individual gastrocnemius muscle protein fractional synthesis. Experimental f: control f ratio for 57 proteins common among each model. The symbols donate a given protein and each group are highlighted by color. For clarity, the proteins are sorted from greatest to least reduction of f in WT-DEX relative to WT-PBS. *C*, Parts of the whole comparison of the percent change in gastrocnemius protein fractional synthesis compared across each group for the 57 common proteins identified. An increase or decrease in f for each protein was determined. A binomial distribution statistic was used to calculate the significance of the number of gastrocnemius muscle proteins with either higher or lower mean fractional synthesis values. 55/57 (*p* ≤ 0.0001), 48/56 (*p* ≤ 0.0001), 48/56 (*p* ≤ 0.0001) and 37/57 (*p* = 0.016) gastrocnemius muscle proteins were lower in WT-PBS *versus* WT-DEX, WT-PBS *versus* MKO-PBS, MKO-PBS *versus* MKO-DEX, and WT-DEX *versus* MKO-DEX, respectively. DEX, dexamethasone.

To determine how DEX treatment in WT and MKO mice leads to muscle atrophy, muscle proteome–wide fractional synthesis rates were measured by LC-MS/MS after ^2^H_2_O labeling. Mice were administered DEX or PBS for 10 days followed by ^2^H_2_O labeling in drinking water for the last 7 days (23, 24, 25, 26). GA muscle protein fractional synthesis criteria for each group (n ≤ 3) filtered the data set to 57 proteins common among each group. Detailed filtering criteria was further explained in the method section (23). Database for Annotation, Visualization, and Integrated Discover ontology analysis was employed to characterize the biochemical function and cellular localization for the proteomics data set. The proteins were grouped as either glucose metabolism (n = 16), mitochondrial (n = 16), cytoplasmic (n = 20), and myofibril proteins, (n = 5) of which, 19 individual proteins were significantly decreased by DEX after Bonferroni correction for multiple comparisons. (Supporting Information 1). Global average GA muscle protein fraction synthesis rates are shown in WT and MKO mice GA muscle treated with PBS or DEX (represented as WT+PBS, WT+DEX, MKO + PBS and MKO + DEX, respectively, Fig. 4*A*). The mean GA protein fraction synthesis values were 18.0%, 13.5%, 15.2%, and 12.9% for WT+PBS, WT+DEX, MKO + PBS and MKO + DEX mice, respectively. The overall protein synthesis rate in MKO mouse GA muscle was significantly lower than that of WT mouse GA muscle (15.5% reduction (15.2%/18.0%), *p* ≤ 0.0001). DEX significantly reduced protein synthesis in both WT and MKO mice. However, in WT mouse GA muscle, the overall protein synthesis rate was decreased by an average of 25.1% (13.5%/18.0%), whereas in MKO mouse GA muscle, the overall protein synthesis rate was reduced by only 15.1% (12.9%/15.2%) with DEX treatment. The relative reduction in the change in GA muscle individual protein fractional synthesis rates were also compared among groups. Experimental fractional synthesis (f) values for the proteins common among each model were plotted in dot plot and sorted from low to high, and then by protein (Fig. 4*B*). Graphically, in WT+DEX (*red line*), MKO + PBS (*green line*), and MKO + DEX mice (*purple line*), most proteins exhibited a decrease in fractional synthesis as compared with WT (*blue line*). We then compared the percent change in each protein’s fractional synthesis for WT animals treated with or without DEX (Fig. 4*C*). Statistical significance was assessed by a binomial distribution of the proportion of proteins showing a negative or positive percent change in fractional synthesis with DEX treatment (Fig. 4*C*). In WT+DEX, 96.5% or 55/57 proteins showed lower fractional synthesis rates as compared with WT mice (Fig. 4*C*). Only two proteins, electron transfer flavoprotein subunit beta and peroxiredoxin-1, did not have lower fractional synthesis by DEX treatment (Supporting Information 1). Surprisingly, in MKO animals, 48/57 or 84.2% of GA muscle proteins showed lower fractional synthesis compared with WT mice (Fig. 4*C*). In addition, 48/57 or 84.2% of proteins in MKO mice were reduced by DEX treatment. Thus, seven proteins whose protein synthesis rates were reduced in WT mice by DEX were not affected by DEX in MKO mouse GA muscle, whereas two proteins, alpha-enolase and phosphoglycerate kinase-1, had a higher protein synthesis rate (17% and 9.4%, respectively) in MKO mice treated with DEX (Supporting Information 2). Finally, 37/57 or 64.9% of GA muscle proteins had lower fractional synthesis in WT + DEX mice compared with MKO + DEX mice, whereas 35.1% had higher fractional synthesis (*p* = 0.016). Overall, these results indicated that DEX effect on lowering protein synthesis was compromised without Pik3r1 in GA muscle.

### Gastrocnemius muscle histology in WT and MKO mice

**Figure 5:**
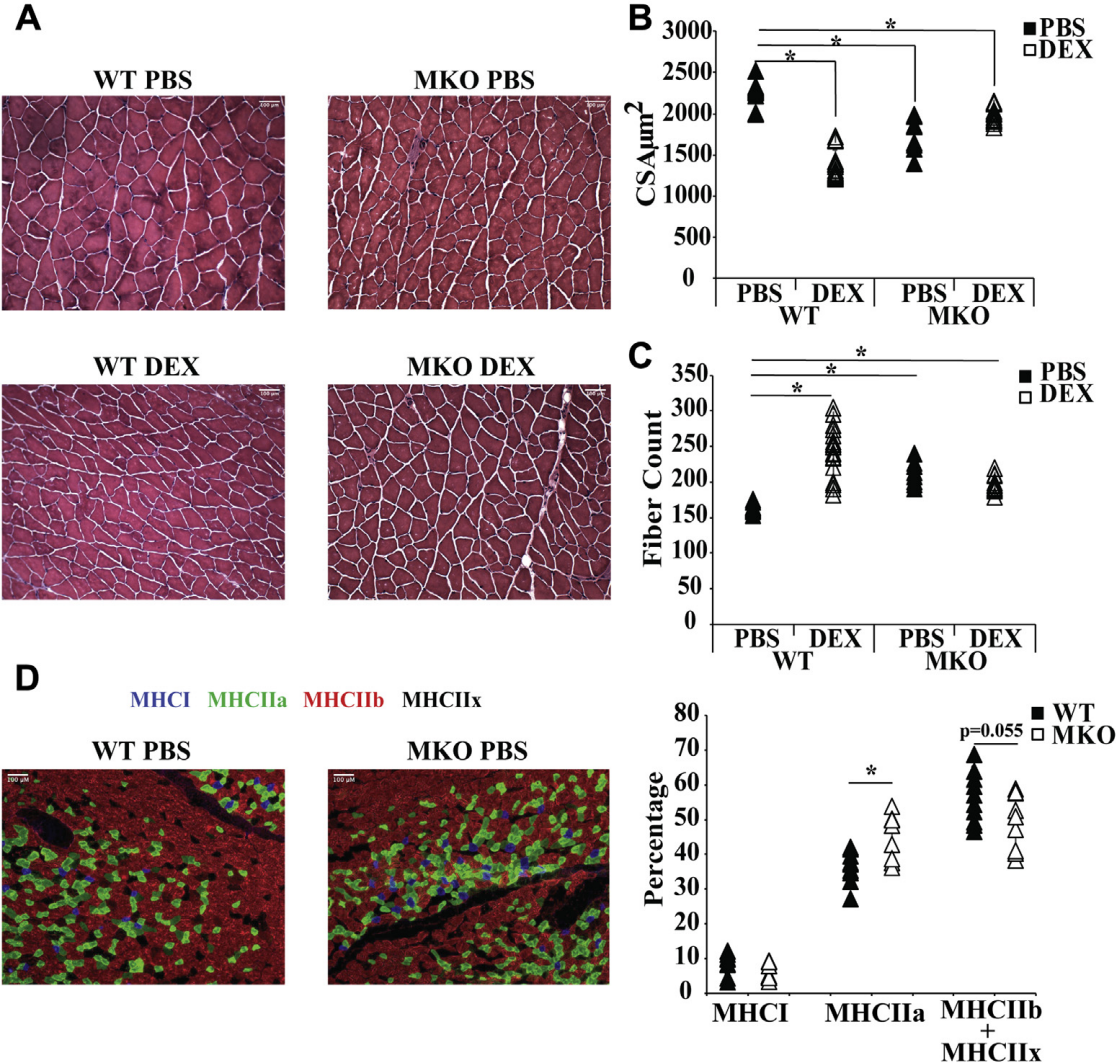
**Analysis of cross-sectional area and fiber number and immunochemistry of gastrocnemius muscle of WT and MKO mice**. *A*, hematoxylin and eosin staining of gastrocnemius muscle of following mice treated with PBS or DEX for 10 days: WT PBS (n = 5), WT DEX (n = 5), MKO PBS (n = 3), and MKO DEX (n = 4). Bar is 100 μm. *B*, cross sectional area and *C*, fiber count were quantified with image J. Statistical differences calculated with an unpaired Student *t* test as compared with only the WT-PBS animals (n = 5). Error Bars represent SD, significance was denoted as ∗*p* ≤ 0.05. *D*, immunohistochemical staining was performed on gastrocnemius muscle of WT PBS and MKO PBS mice using antibodies of MHCI, MHCIIa, MHCIIb, and MHCIIx. All fibers in each picture were counted and then calculated the percentage of each fiber type in the total number of fibers. For WT PBS, 12 pictures were counted whereas for MKO PBS 10 pictures were counted. Bar is 100 μm. Error bars represent SD, significance denoted as ∗*p* ≤ 0.05. DEX, dexamethasone.

We also performed histological analysis (Fig. 5*A*) to monitor the cross-section area and fiber count of GA muscle of WT + PBS, MKO + PBS, WT + DEX, and MKO + DEX mice, asking whether DEX induced muscle histological changes are compromised in MKO mice. Cross-sectional area of GA muscle fibers significantly decreased from a mean of 2277 μm^2^ in WT animals to 1382 μm^2^ in WT + DEX mice (Fig. 5*B*). In MKO mice, cross-sectional area of GA muscle fibers was 1709 μm^2^ (Fig. 5*B*). This is in agreement with the lower protein synthesis rates in MKO GA muscle shown above. Importantly, there was no reduction in cross-sectional area of GA muscle fibers in MKO + DEX mice (1979 μm^2^) compared with MKO mice. GA muscle fiber count showed similar results. DEX treatment increased fiber count in WT GA muscle, and MKO had higher fiber count than WT mice, but DEX did not alter fiber count in MKO mice (Fig. 5*C*).

Immunohistochemical staining was used to analyze the fiber types of GA muscle of WT and MKO mice. The antibodies against MHC I, MHC IIa, MHC IIb, and MHC IIx were used. MHC I is encoded by Myh7 and represents slow oxidative fibers, whereas MHC IIa is encoded by Myh2 and represents fast oxidative fibers (27). MHC IIb is encoded by Myh4, and MHC IIx is encoded by Myh1 and represent fast glycolytic fibers IIb and IIx, respectively (27). The fiber type composition of GA muscle of WT mice was similar to previous reports (Fig. 5*D*) (28). It appears that GA muscle of MKO mice had more fast oxidative fibers, as MHC IIa levels were higher than those of WT mice (Fig. 5*D*).

### DEX effect on signaling pathways regulating protein synthesis

**Figure 6:**
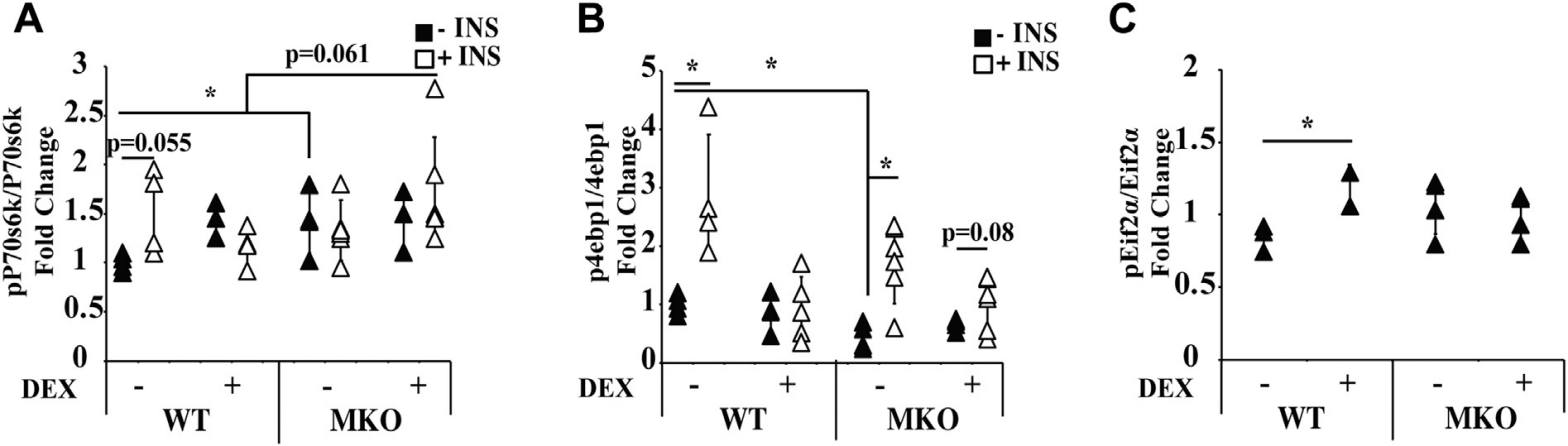
**Comparing signaling pathways that regulate protein synthesis in gastrocnemius muscle of WT and MKO mice**. WT and MKO mice were treated with or without DEX (10 mg/kg body weight) for 1 week. At the end of treatment, mice were IP injected with insulin (1 unit per kg body weight) or PBS for 10 min, and gastrocnemius muscle was isolated and processed for ELISA to monitor the phosphorylation status of (*A*) p70S6K at Thr389, (*B*) 4E-BP1 at Thr37/Ser46 and (*C*) eIF2α at Ser52. n = 3 to 6, significance was denoted as ∗*p* ≤ 0.05. Error bars represent the SD. DEX, dexamethasone.

We further analyzed the DEX effect on signaling processes that regulate protein synthesis. As described above, WT and MKO mice were treated with or without DEX and were injected with insulin for 10 min before GA muscle was isolated. We first monitored the phosphorylation at threonine 389 of p70S6 kinase (S6K) (29), which is critical for S6K activity, using ELISA. S6K phosphorylates the S6 protein of the 40S ribosomal subunit and is involved in translational control of 5' oligopyrimidine tract mRNAs (29). We found that insulin treatment increased the levels of Thr389 of S6K in WT mice (Fig. 6*A*). DEX treatment abolished this insulin induction (Fig. 6*A*). Interestingly, in MKO mice, neither insulin nor DEX affected S6K Thr389 phosphorylation (Fig. 6*A*).

Next, we monitored the phosphorylation of threonine 37 and 46 of 4E-BP1 (30). 4E-BP1 is a translational repressor that inhibits cap-dependent translation by binding to the translation initiation factor eIF4E (31). Phosphorylation at Thr37 and Thr46 is required for subsequent phosphorylation at serine 65 and threonine 70, which are critical for 4E-BP1 function (30, 32). We found that insulin induced 4E-BP1 phosphorylation in WT mice, whereas DEX treatment abolished such effect (Fig. 6*B*). Interestingly, the basal levels of 4E-BP1 phosphorylation were lower in MKO mice (Fig. 6*B*). Insulin still increased 4E-BP1 phosphorylation in MKO mice (Fig. 6*B*). DEX treatment reduced such insulin effect (Fig. 6*B*). However, unlike in WT mice, DEX treatment did not abolish insulin effect. Thus, without Pik3r1, some degrees of the insulin effect on 4E-BP1was restored (Fig. 6*B*).

Finally, we monitored the phosphorylation at serine 52 of translational initiator eIF2α. eIF2 consists of three subunits, eIF2α, eIF2β, and eIF2γ, that promotes a new round of translation initiation by exchanging GDP for GTP, which is catalyzed by eIF2B (33, 34). The phosphorylation of eIF2α stabilizes the eIF2–GDP–eIF2B complex that inhibits the turnover of eIF2B to attenuate the translation (33, 34). This phosphorylation is not regulated by insulin. Thus, we just compared the phosphorylation status of eIF2α in WT and MKO mice treated with or without DEX. Interestingly, DEX treatment elevated eIF2α phosphorylation in WT but not MKO mice (Fig. 6*C*). The basal eIF2α phosphorylation was similar between MKO and WT mice (Fig. 6*C*). However, in contrast to the DEX effect in WT mice, DEX treatment did not affect eIF2α phosphorylation in MKO mice (Fig. 6*C*).

In summary, there are three major observations from these results. First, in GA muscle, DEX inhibited insulin induced p70S6K and 4E-BP1 activity. Moreover, DEX treatment increases eIF2α phosphorylation. All these responses could contribute to the suppression of protein synthesis. Second, without Pik3r1, the basal 4E-BP1 phosphorylation was decreased. This phenomenon could contribute to the lower protein synthesis rate in GA muscle of MKO mice. Third, without Pik3r1, the ability of DEX to suppress insulin’s effect on 4E-BP1 was impaired, and DEX was unable to potentiate eIF2α phosphorylation. These results could explain the partial maintenance of protein synthesis rates in GA muscle of MKO mice.

## Discussion

Glucocorticoids modulate protein and glucose metabolism through suppressing insulin signaling in skeletal muscle (1, 2). The mechanisms governing this process, however, are not completely understood. GR is a transcription factor, and its primary target genes initiate the physiological responses of glucocorticoids. We previously identified *Pik3r1* as a GR primary target gene that mediates glucocorticoid responses in murine C2C12 myotubes. In this study, we further demonstrated that the *Pik3r1* gene is activated by GR in mouse GA muscle, because GR was recruited to the *Pik3r1* GRE, and the levels of histone acetylation were elevated upon DEX treatment. GR was also recruited to the *Pik3r1* GRE, and there were significant levels of acetylated H3 and H4 at the *Pik3r1* GRE in PBS-treated mice. These results suggest that physiological corticosterone levels at the time we performed ChIP contribute the basal expression of *Pik3r1* in mouse GA muscle. As plasma corticosterone levels are modulated by circadian rhythm, we predict that the degree of GR recruitment to the Pik3r1 GRE could vary depending on the time we conduct ChIP experiments. Notably, even during the refeeding stage when plasma corticosterone levels are low, GR is recruited to certain GREs (35). However, such recruitment is usually much weaker than times of high plasma corticosterone levels, such as fasting (35).

While Pik3r1 serves as a regulatory subunit for PI3K, a key signaling molecule in insulin signaling, previous studies have shown that overexpression of Pik3r1 reduces whereas depletion of Pik3r1 enhances insulin action in metabolic tissues, such as skeletal muscle, white adipose tissue, and liver (10, 11, 12, 13, 36). Human genomic studies have associated *Pik3r1* to insulin resistance (37, 38, 39). In this study, we further demonstrated the role of Pik3r1 in glucocorticoid-induced glucose and insulin intolerance *in vivo*. We found that depletion of Pik3r1 in GA muscle compromised the ability of glucocorticoids to suppress insulin signaling in GA muscle but not in two other insulin responsive metabolic tissues, epididymal white adipose tissue, and liver. This likely results in improved glucose and insulin intolerance in DEX-treated MKO mice.

Using the combination of stable isotope labeling and LC/MS-MS technology, we were able to further investigate DEX effects on GA muscle *in vivo* protein turnover (23) and the role of Pik3r1 in this process. Not surprisingly, DEX treatment significantly reduced the overall protein synthesis rate (40). Intriguingly, among 57 proteins with kinetics quantified by LC/MS-MS in all four groups, 55 had lower protein synthesis rate with DEX treatment. Ontology analysis showed that these 55 proteins contained enzymes in the glycolysis and TCA cycle, as well as mitochondrial and myofibrillar proteins. The protein synthesis rate suppression by DEX treatment was significantly attenuated upon the depletion of Pik3r1. Not surprisingly, the DEX suppressed protein synthesis rate was not entirely abolished by the depletion of Pik3r1, as other GR primary target genes, such as *MuRF1* (a.k.a. *Trim63*) and *Ddit4*, are known to play a role in this process (40, 41). The results from GA skeletal muscle cross-section area, and fiber counts are consistent with and complement the protein synthesis data, revealing decreased myotube diameters in DEX treated WT and in MKO mice, but no further reduction by DEX treatment in MKO mice.

Our study was not designed to investigate the effects of glucocorticoids on protein degradation. Measurement of protein breakdown by *in vivo* metabolic labeling is best determined by analyzing replacement (turnover) rates at steady-state, where synthesis is balanced by breakdown so high turnover rates and a lower protein pool size can be taken to reflect stimulated breakdown, even in the presence of reduced absolute synthesis rates (23, 42). Our studies here were carried out during the early phase of decreasing skeletal muscle mass after DEX treatment, not during steady state, so it is problematic to infer breakdown rates. Accordingly, our findings document globally lower protein synthesis rates during the 10 days after initiation of DEX treatment but do not rule out a contribution from higher protein breakdown rates as well (1, 40, 43, 44, 45, 46).

One surprising result is that the basal protein synthesis rate in MKO mice GA muscle was significantly lower than that of WT mice. Thus, Pik3r1 appears to play a role in basal protein synthesis that is in addition to its role in glucocorticoid action. Our signaling studies found that the basal phosphorylation of 4E-BP1 was lower in GA muscle of MKO mice than in WT mice. This observation might explain, at least in part, a lower protein synthesis rate in GA muscle of MKO mice. GA muscle of MKO mice also contained high percentages of fast oxidative fibers, which could be the reason for smaller fiber sizes of GA muscle in MKO mice. Notably, in GA muscle of MKO mice, insulin failed to enhance p70S6K. However, we do not think that this contributes to a lower protein synthesis rate in MKO mice, as pp70S6K/p70S6K ratio was similar between WT and MKO mice that were treated with insulin. It is not surprising that DEX treatment inhibited insulin-induced p70S6K phosphorylation in WT mice. However, it is unclear why p70S6K was not induced by insulin in MKO mice, whereas its upstream kinase Akt was stimulated by insulin in MKO mice. More detailed studies are needed to explain this observation.

The regulation of 4E-BP1 by insulin and DEX was somewhat similar to their regulation of its upstream kinase, Akt, in MKO mice (Fig. 3*A*). Thus, in Pik3r1-depleted GA muscle, the ability of DEX to suppress insulin increased 4E-BP1 phosphorylation was decreased. We also found that DEX treatment increased eIF2α phosphorylation in WT mice GA muscle, which was in agreement with a reduced protein synthesis rate. This DEX effect was not observed in MKO mice. These results could explain a partially restored protein synthesis rate in GA muscle of MKO mice. Interestingly, the induction of eIF2α phosphorylation by DEX was not found in previous studies (47, 48). In one report, eIF2α phosphorylation was analyzed in GA muscle of male Sprague-Dawley rats that were IP injected with DEX for 4 h (47). In another report, eIF2α phosphorylation was examined in biopsies that were conducted in young health male volunteers taking oral DEX every 6 h for 3 days (48). In addition to species difference, our treatment conditions were distinct to theirs, as we treated mice with DEX for 1 week. It is possible that a longer DEX exposure results in the activation of eIF2α kinase(s), such as PKR, PERK, GCN2, and/or HRI (49, 50). This notion will need to be tested in future.

The role of Pik3r1 in DEX effects on eIF2α phosphorylation and maintaining proper proportions of fast oxidative fibers in GA muscle are likely independent of its function in insulin actions. Pik3r1 has been shown to participate in cellular functions that are independent of its role in insulin action. For example, Pik3r1 is involved in glucocorticoid-induced lipolysis in adipose tissue by increasing PKA signaling in the lipid droplet (51). Moreover, Pik3r1 has been shown to participate in trafficking of receptor tyrosine kinases and the erythropoietin receptor (52, 53) and is required for the nuclear localization of XBP1 (37, 54). How Pik3r1 acts in basal and DEX reduced protein synthesis rates in skeletal muscle requires further studies.

In conclusion, we demonstrate for the first time that Pik3r1 is involved in glucocorticoid inhibition of insulin signaling and protein synthesis rate in GA muscle *in vivo*. Moreover, Pik3r1 plays a role in maintaining basal protein synthesis rates and the proper proportion of fast oxidative fibers in murine GA muscle.

## Experimental procedures

### Animal subjects

Mice with a conditional allele of Pik3r1 gene flanked with LoxP sites at exon7 (*Pik3r1*^*flox/flox*^, will be referred as WT, WT, mice) were provided by the laboratory of Lewis Cantley (Weill Cornell Medical College) (55). Muscle-specific Pik3r1 knockout mice (MKO) were generated by crossing *Pik3r1*^*flox/flox*^ with transgenic mice expressing Cre recombinase under control of muscle creatine kinase (B6.FVB(129S4)-Tg(Ckmm-cre)5Khn/J) (20). The following primers were used for genotyping: Pik3r1_loxP_F (CACCGAGCACTGGAGCACTG), Pik3r1_loxP_R (CCAGTTACTTTCAAATCAGCACAG), AdipoQ_Cre_F (GCGGTCTGGCAGTAAAAACTATC), AdipoQ_Cre_R (GTGAAACAGCATTGCTGTCACTT), Ckmm_Cre_F (TAAGTCTGAACCCGGTCTGC), Ckmm_Cre_R (GTGAAACAGCATTGCTGTCACTT). In AKO mice, ∼310 bps amplified by Pik3r1_loxP_F and Pik3r1_loxP_R primers and ∼100 bps amplified by AdipoQ_Cre_F and AdipoQ_Cre_R primers were observed. In MKO mice, ∼310 bps amplified by Pik3r1_loxP_F and Pik3r1_loxP_R primers and ∼500 bps amplified by Ckmm_Cre_F and Ckmm_Cre_R primers were observed. In *Pik3r1*^*flox/flox*^ (WT) mice only ∼310 bps amplified by Pik3r1_loxP_F and Pik3r1_loxP_R primers were observed. Eight-week-old male MKO and WT mice were injected intraperitoneally with 5 or 10 mg/kg body weight of DEX (water soluble dexamethasone, Sigma D2915) or PBS (control) for 1, 4, or 7 days. At the end of the treatment period, blood, inguinal, and epididymal adipose tissues, liver, and GA muscle were isolated from mice for protein expression analysis. Mice were housed in ventilated cages with Sanichip bedding along with a cotton Nestlet and a 4gm puck of crinkled paper. They were co-housed and were fed a diet of 18% protein, 6% fat (Envigo 2918) with a 12-h light and dark cycle in a temperature-controlled room of approximately 22 °C. The Office of Laboratory Animal Care at the University of California, Berkeley (AUP-2014-08-6617) approved all animal experiments conducted in this work.

### Western blot

The protein concentration for samples was measured with Bradford protein dye (BioRad). Proteins (∼30 μg) were mixed with 1× NuPAGE LDS Sample Buffer (ThermoFisher, NP0007) and 1× NuPAGE Sample Reducing Agent (ThermoFisher, NP0009), boiled for 5 min before being applied to SDS-PAGE. The following are the antibodies we used in this study: anti-Gapdh (Santa Cruz, sc-25778), anti-Pik3r1 (Cell Signaling, 4292s). The intensity of the bands was quantified using Image J software (Rapsand NIH, 1997–2018) and normalized to Gapdh.

### Chromatin immunoprecipitation

WT mice were intraperitoneally injected with 10 mg/kg body weight of DEX (water soluble DEX, Sigma D2915) for 4 days. On the last day, GA muscles were harvested and snap frozen with liquid nitrogen. Frozen muscles were ground to fine powder with pestle. Then, tissue powder was cross-linked with 1% formaldehyde in 20 ml PBS at 37 °C for 10 min with gentle shaking. After quenching the cross-linking reaction with 125 mM glycine, samples were centrifuged at 1000*g*, 4 °C for 5 min. Pellets were washed with ice-cold PBS, then resuspended in 3 ml buffer S (50 mM Tris pH 8.0, 1% SDS, 10 mM EDTA, 1 mM DTT, 100 mM MG 132, and protease inhibitor cocktail). Samples were incubated on ice for 10 min, then sonicated with the Branson Sonifier 250 sonicator for 50 s (60% output, 10 s pulse with 40 s rest). After centrifugation for 10 min at 32,000*g*, 4 °C, supernatant containing sheared DNA fragments, was collected, and mixed with one sample volume of buffer D (0.01% SDS, 1.1% Triton x-100, 1.2 mM EDTA, 16.7 mM Tris [pH 8.0], 167 mM NaCl, 100 mM MG132 and a protease inhibitor cocktail). Diluted sample was then incubated with 100 μl of 50% protein A/G agarose beads (sc-2003, Santa Cruz) for 1 h at 4 °C with gentle shaking to preclean the sample. After centrifugation at 4000*g* for 3 min at 4 °C to pellet the agarose beads, supernatant was used to set up the IP reactions. The following antibodies were used in this study: anti-IgG (sc-2027, Santa Cruz), anti-GR (sc-393232, Santa Cruz), anti-H3 histone (ab1791, abcam), anti-H4 (05-858, Millipore), anti-AcH3 (ab47915, abcam), anti-AcH4 (06-866, Millipore), anti-H3K4me3 (ab8580, abcam), anti-H3K4me1 (ab8895, abcam), and anti-p300 (sc-584x, Santa Cruz). Samples were allowed to react with antibody for 18 h (overnight incubation) at 4 °C with gentle shaking. Then, 50 μl of 50% protein A/G agarose beads were added into each IP reaction and were rotated for 2 h at 4 °C. Then, agarose beads were washed with the following conditions: 1x low-salt wash buffer (0.1% SDS, 1% Triton X-100, 2 mM EDTA, 20 mM Tris [pH 8.0] and 150 mM NaCl), 1x high-salt wash buffer (0.1% SDS, 1% Triton X-100, 2 mM EDTA, 20 mM Tris [pH 8.0], and 500 m NaCl), 1x LiCl wash buffer (0.25 M LiCl, 1% NP-40, 1% sodium deoxycholate, 1 mM EDTA and 10 mM Tris [pH 8.0]), and 2x Tris-EDTA buffer. After the last wash, all supernatants were removed, then 400 μl of elution buffer (10 mM DTT, 1% SDS and 0.1 M NaHCO3) was added. Samples were rotated at room temperature for 1 h, then centrifuged at 8000*g* for 1 min. Supernatant was transferred to a new tube and were mixed with 16 μl of 5 M NaCl and were incubated at 65 °C overnight. On the last day, 16 μl of Tris (pH 6.5), 8 μl of 0.5 M EDTA, and 1.5 μl of proteinase K (EO0491, Thermo Scientific) were added to the sample and were incubated at 55 °C for 3 h. The immune-precipitated DNA fragments were extracted with a PCR clean up kit (28106, Qiagen), then were applied to qPCR to quantify the IP result.

### Intraperitoneal glucose tolerance test

Eight-week-old male MKO and WT mice were treated with 10 mg/kg body weight of DEX in drinking water. DEX was dissolved in PBS, which was then added into the drinking water. For control, equal amount of PBS was added into the drinking water. After a 15 h fast, mice were injected with 1 g/kg body weight glucose for an intraperitoneal glucose tolerance test. Tail vein blood was used to monitor blood glucose levels at different time points: 0 (before glucose injection), 15, 30, 60, 90, and 120 min after glucose injection using a blood glucose meter (Contour, Bayer).

### Insulin tolerance test

Fed mice were injected with 1 unit/kg body weight insulin (Sigma, I0516-5ML) intraperitoneally. Tail vein blood was used to monitor blood glucose levels at different time points: 0 (before glucose injection), 15, 30, 60, 90, and 120 min after glucose injection using a blood glucose meter (Contour, Bayer).

### Plasma insulin analysis

Plasma insulin levels were examined using an ultrasensitive mouse insulin ELISA kit (Crystal Chem Inc, Cat. No: 90080).

### ELISA using tissue homogenates

Mice (as described above) were treated with 10 mg/kg bodyweight of DEX (water-soluble) or PBS (control) for 7 days. After intraperitoneal injection of 1 unit/kg bodyweight insulin for 10 min, mice were euthanized, and GA muscle were collected. The tissue was homogenized in cell lysis buffer (Cell Signaling Technology, #9803 or Multispecies InstantOne ELISA Kit Lysis Buffer [Invitrogen Catalog #85-86046-11]) using the BeadBug 6 Homogenizer (Benchmark Scientific.) Homogenized lysate was centrifuged at 13,300 rpm for 15 min at 4 °C. Supernatant was transferred into fresh tubes and used for the following ELISAs.

### Akt and pAkt ELISA

Relative Akt and pAkt levels were measured using the Akt (Total/Phospho) Multispecies InstantOne ELISA kit (Invitrogen, Catalog #85-86046-11.) Tissue lysate were loaded with a protein concentration of 0.5 μg/μl. All steps afterward were followed according to manufacturer’s instructions.

### S6 kinase and p70S6 kinase ELISA

Relative S6 kinase and p70S6 levels were measured using the p70 S6 Kinase (Total/Phospho) Multispecies InstantOne ELISA Kit (Invitrogen, Catalog #85-86053-11.) Tissue lysates were loaded with a protein concentration of 1 μg/μl. All steps afterward were followed according to manufacturer’s instructions.

### 4E-BP1 (total and phosphorylated) ELISA

Relative total 4E-BP1 and phosphorylated 4E-BP1 levels were measured using the Phospho-4E-BP1 (Thr36) and Total 4E-BP1 ELISA kit (RayBiotech, Catalog #PEL-4EBP1-T36-T). Tissue lysates were loaded with a protein concentration of 1.25 μg/μl and 1 μg/μl. All steps afterward were followed according to manufacturer’s instructions.

### eIF2α and phospho-eIF2α ELISA

Relative total eIF2α and phosphorylated eIF2α levels were measured using the PathScan Phospho-eIF2α (Ser51) and total eIF2α Sandwich ELISA kits (Cell Signaling Technology, Catalog #7286 and Catalog #7952.) Tissue lysates were loaded with a protein concentration of 0.25 μg/μl.

### Chemicals and reagents

All reagents were purchased or made with the highest quality experimental conditions. 1x PBS (Gibco), hyclone molecular grade water (GE healthcare), 0.5 M EDTA (Gibco), 0.1 M PMSF (Sigma), Halt protease inhibitor (78429, Thermo scientific), 2,2,2-triflouroethanol (Sigma), ammonium bicarbonate (Sigma), DTT (Sigma), IAA (Sigma), trypsin (Sigma), acetonitrile (Fisher), formic acid (Fisher), and deuterium oxide (Cambridge isotope labs).

### ^2^H_2_O labeling protocol

Mice were administered 99.0% sterile deuterium oxide (^2^H_2_O) (Cambridge isotope laboratories) at the third day of DEX treatment *via* intraperitoneal injection. The animals remained on DEX and ^2^H_2_O for seven more days until time of sacrifice. Animals were maintained on a maintenance dose of 8.0% ^2^H_2_O in drinking water to maintain approximately 5.0% excess ^2^H enrichment in total body water (24).

### Tissue harvesting

Mice were euthanized with 2 l/min flow rate of CO_2_ followed by cervical dislocation. Blood was collected *via* cardiac puncture and was centrifuged at 12,000*g* for 10 min to obtain plasma and stored at −20 °C. GA muscle samples were flash frozen with liquid nitrogen and stored at −80 °C until processing.

### Histology and cross-sectional fiber determination

Whole GA muscle was carefully dissected, placed in a cryomold with an optimal cutting temperature solution and was flash frozen in a liquid nitrogen cooled metal block. Ten micrometer transverse muscle sections were performed using a cryotome at −20 °C, followed by transfer to positively charged microscope slides for hematoxylin and eosin staining. Muscle fiber diameter was recorded using a light microscope, and cross-sectional area was analyzed *via* Image J (Rapsand NIH, 1997–2018).

For immunohistochemistry (56, 57, 58), 10 μm transverse sections from the midportion of the GA muscles were obtained. Slides were air dried for 10 min followed by washing with PBS + 0.05% Triton X (PBST) for 10 min and then for 5 min. Next, sections were blocked in PBST containing 10% goat serum for 1 h at room temperature and then incubated overnight at 4 °C with a mixture of three primary mouse monoclonal antibodies (MYH7 (BA-F8, IgG2b), MYH2 (SC-71, IgG1), and MYH4 (BF-F3, IgM), obtained from DSHB at the University of Iowa) in PBST containing 10% goat serum. Next day, slides were washed with PBST for 10 min and then with new PBST for 5 min. After washes in PBST, sections were incubated for 1 h with a mixture of three goat anti-mouse secondary antibodies against IgG2b (Alexa 350), IgG1 (Alexa 488), and IgM (Alexa 555) in 10% GS/PBST, followed by washes with PBST for 10 min and then with new PBST for 5 min. Sections were then mounted with Fluromount-G, SouthernBiotech, and sealed with nail polish. Muscle fibers positive for each MHC isoform were counted manually using Image J and presented as a percentage of fiber types.

### Measurement of heavy water body enrichment using acetone exchange and GC/MS

Heavy body water enrichment was analyzed by distillation of water from plasma followed by acetone exchange and was measured by GC/MS (23). Deuterium enrichment was used in the calculation of fractional synthesis values based on mass isotopomer distribution analysis (MIDA) (25). This enrichment tells us the maximal theoretical enrichment of each particular isotopomer at a given exposure to ^2^H_2_O. This maximal enrichment, representing 100% newly synthesized protein molecules, is then compared to the experimentally measured value to determine the fractional synthesis of the protein based on the rise to plateau precursor-product relationship (25). One hundred microliter of mouse plasma was distilled overnight at 80 °C. Deuterium labeled water was exchanged with acetone in an alkaline environment by incubating 1.0 μl of 10.0 M NaOH and 5.0 μl of acetone overnight at room temperature. Three hundred microliter of hexane was added followed by removal of the organic layer to another tube with sodium sulfate. The hexane sample with deuterium enriched acetone was then injected into the GC/MS. Unlabeled M0 and labeled M1 was quantified by integrating the area under the curve. A standard curve of percent enrichment as a function of the M1 ratio was simultaneously generated during sample processing. A quadratic equation was used to measure the percent enrichment of the unknown sample based on the given M1 ratio. Body water enrichments were used as the precursor pool enrichment for determining protein product enrichments based on MIDA calculations (23, 24).

### In solution digest for dynamic proteomics

Dynamic proteomic technologies developed in our laboratory was used to determine how DEX treatment and/or MKO affected the turnover of GA muscle protein synthesis rates (23, 24). This approach is able to measure the synthesis rates of large numbers of proteins across the proteome in a single experiment that combines tandem mass spectrometry with stable isotopic metabolic labeling with ^2^H_2_O. Approximately 100 mg of GA muscle underwent an in-solution digest protocol (23). Briefly, 500 μl of 1× PBS, 1 mM PMSF, 5 mM EDTA, 1X halt protease inhibitor was added to each sample, homogenized with Qiagen tissue lyser at 30 Hz for 2.0 min, then centrifuged at 10,000 RPM for 10 min at 4 °C. Two hundred and fifty microgram of supernatants were quantified with a bicinchoninic acid assay (Pierce BCA Protein Assay Kit) and speed vacuumed until completely dried. Twenty five microliter of 2,2,2-trifloroethanol, 25 μl of 100 mM ammonium bicarbonate, and 2.3 μl of 100 mM DTT were added to each sample and incubated at 60 °C for 1 h, followed by 10 μl of 100 mM of IAA and incubated at room temperature (RT) for 1 h in the dark. 2.0 μl of 100 mM DTT was added to remove residual IAA, incubated for 20 min at RT, and diluted with 100 μl of 100 mM ammonium bicarbonate and 300 μl of pure water. 1:50 of trypsin to protein ratio was used for overnight digest at 37 °C, and 5.0 μl of formic acid was used to stop digest. Samples were concentrated with a speed vac until ∼300 μl, centrifuged at 10,000 RPM for 30 min. Supernatants were cleaned up with a C18 SPEC tip, speed vacuumed dried, and submitted in 30 μl of 3.0% acetonitrile/0.1% formic acid LC-MS submission buffer.

### Experimental design and statistical rationale for LC-MS/MS analysis of proteome dynamics

To determine how DEX treatment in WT and MKO mice leads to muscle changes, we measured GA muscle protein fractional synthesis rates using shot-gun LC-MS/MS analysis after *in vivo* ^2^H_2_O labeling (23, 24, 26). Mice were treated with PBS or DEX for 10 days. During the final 7 days, mice were also labeled with ^2^H_2_O. The sample size for WT controls treated with PBS (WT-PBS, n = 6), WT treated with DEX (WT-DEX, n = 6), MKO treated with PBS (MKO-PBS, n = 5), and MKO treated with DEX (MKO-DEX, n = 5). The rational for biological replicates of n = 5 to 6 were based on prior data for biologic and analytic variability of proteome-wide protein fluxes (23, 24, 26). Biological replicates were chosen to assess biological variability rather than sole reliance on technical replicates. Controls included WT-PBS treated mice (n = 6). Two separate dynamic proteomic experiments were completed and combined for final data analysis. This consisted of experiment 1 that included WT-PBS (n = 4) and WT-DEX (n = 4). Experiment 2 included the final biological replicates of WT-PBS (n = 2), WT-DEX (n = 2), MKO-PBS (n = 5), and MKO-DEX (n = 5). The data sets were combined and annotated as described by the following. We used several filtering criteria for inclusion of protein kinetic data that were included in the comparisons (23, 24): more than one peptide had to be present for all proteins included; each peptide had to meet analytic accuracy criteria for fractional mass isotopomer abundances and for reproducibility among mass isotopomers; a protein had to be present in at least three animals per group; and these criteria had to be met for the protein in all four groups. The number of proteins meeting these criteria was 81 in WT-PBS, 88 in WT-DEX, 124 in MKO-PBS, and 134 in MKO-DEX, and 57 proteins met these criteria in GA muscle in all four groups. To determine the difference in protein fractional synthesis rates (f), four groups were categorized as the following: WT-PBS, WT-DEX, MKO-PBS, and MKO-DEX. The mean, medium, and standard deviation for each protein (n ≥ 3 for each group) were calculated, and a 2X2 ANOVA analysis (InfernoRDN v1.1.6970; January 31, 2019) was performed to compare the treatment, genotype, and interaction effects. Protein fractional synthesis were averaged within groups, and the percent changes were compared across each group. An increase or decrease in fractional synthesis was assessed as ±0.0%. A binomial distribution statistical analysis was used to calculate the significance of the relative percent increase or decrease in GA protein fractional synthesis. Average protein fractional synthesis was also assessed by ANOVA followed by the Benjamini and Hochberg test for multiple comparisons (false discovery rate [FDR] = 0.05, *p* ≤ 0.05) using GraphPad Prism version 8.0 for Mac, GraphPad Software.

### Search parameters and acceptance criteria (MS/MS and/or peptide fingerprint data)

MassHunter release, version B.07.00, was the software used for peak list generation. The search engine for proteomic analysis based on MS/MS identifications was Spectrum Mill released version B.04.01. Uniprot (2018) was the sequence database searched for mouse protein identifications (59). The overall number of entries searched in the data base were 17,019. Specificity of all proteases included conical tryptic cleavage site of the C-terminal side of lysine and arginine amino acid residues. The number of missed cleavages permitted were two. Fixed modifications included cystines carbamidomethylation (C). Variable modifications included acetylated lysine (K), oxidized methionine (M), N-terminal pyroglutamic acid (N-termQ), deamidated asparagine (N), and hydroxylated prolines (P). Mass tolerance for precursor ions and fragment ions were 20 ppm and 30 ppm, respectively. For accepting individual spectra, the threshold score/expected value was 30% based on the minimum match peak intensity. Estimation of global FDR was 1.0% that was determined by algorithms of the Spectrum Mill software and validated at the peptide and protein levels.

### LC-MS proteomics and mass isotopomer kinetic analysis

Comprehensive reviews of proteome dynamics have previously been described in detail (23, 24). Briefly, the distribution of deuterium labeled peptides for determining GA muscle protein replacement rates was separated, analyzed, and quantified using Agilent 6550 LC/MS-MS equipped with chip cube nano ESI source and quadrupole time of flight mass analyzer (Agilent technologies). HPLC separated the complex mixture using capillary and nano binary flow. Mobile phase consisted of 3.0% acetonitrile (v/v) and 0.1% formic acid in LC/MS grade water (buffer A) to 95% acetonitrile, 0.1% formic acid in LC/MS grade water. Samples were enriched on the chip cube enrichment column with buffer A for 18 min and were eluted with an increasing gradient with final percentages of 95.0% acetonitrile to 5.0% water for 12 min, and columns were finally equilibrated with buffer A for 10 min. Samples were injected once, and mass spectra were collected in MS/MS mode. Peptide isotope ratios and abundances were collected in MS mode. MS/MS data were analyzed with Agilent Spectrum Mill software B.04.01 and Swissprot/Uniport (2018) database for protein identification against the mouse protein database (59). The mass isotopomer patterns that contain the kinetic information in the MS spectrum for each peptide was extracted using Agilent Mass hunter B.07.00 analysis software (24). Filtering criteria included a false discovery rate of 1.0% and ±5.0% of the predicted isotopomers distribution, and baseline abundance of 30,000 counts. Peptide elemental composition was calculated from peptide sequence. Formula, mass, and retention time for a particular peptide was used to obtain mass isotope abundances from the MS files. Mass isotopomer distribution patterns for kinetic measurements were determined from precursor body water enrichments (p), number deuterium label (n) amino acid from active metabolic labeling with ^2^H from heavy water. MIDA was employed using programs developed by Hellerstein *et al.* to determine the fractional synthesis of the parent protein (23, 26) for each peptide based on the isotopomer distribution pattern and enrichments of the labeled tryptic peptides. The labeled tryptic peptides were analyzed by LC/MS-MS to obtain the fractional synthesis rates (change in enrichment of M_0_ isotopomers at time (t)/asymptomatic enrichment of M_0_ isotopomer as predicted by MIDA) of the parent protein and calculated from the rounded up values of its labeled peptides (24, 26). The data analysis was handled with Microsoft excel (Version 16.28), Prism V8.0, and InfernoRDN (v1.1.6970; January 31, 2019). Gene ontology analysis was completed with the Database for Annotation, Visualization, and Integrated Discover for pathway analysis (60, 61). Proteins were then grouped in functional categories such as glycolytic, mitochondrial, cytosolic, and myofibrillar proteins.

## Data availability

Data can be accessed publicly online *via* Figshare here: https://figshare.com/projects/The_role_of_striated_muscle_Pik3r1_in_glucose_and_protein_metabolism_following_chronic_glucocorticoid_exposure/82868. Project ID is 82868. One can access raw mass spectrometry data for identifications and mass isotopomer distribution analysis protein kinetic data as well. Supporting information tables can be found in Supporting Information 1. They contain protein identification, uniprot accession number, number of distinct peptides assigned for each protein, percent coverage, and quantification measurements for each protein for identification and kinetic analysis.

## Statistics and gene ontology

Dynamic proteomics statistics analysis was explained in the section above. Data are expressed as standard deviation (SD) for each group and comparisons were analyzed by Student’s *t* test.

## Conflict of interests

The authors declare that they have no conflicts of interest with the contents of this article.
