# Supplementary material for: The role of striated muscle Pik3r1 in glucose and protein metabolism following chronic glucocorticoid exposure
Source: J Biol Chem. 2021 Feb 7;296:100395. doi: 10.1016/j.jbc.2021.100395 (PMC8010618; doi:10.1016/j.jbc.2021.100395)
Supplement: Supporting Information 1 [file mmc7.docx]

**Supporting Information 1**. Fractional synthesis (%) of 57 individual gastrocnemius muscle proteins (n≥3 per group) in wild type and MKO mice treated with PBS or DEX.

| **Protein** | **WT-PBS** | | **WT-DEX** | **MKO-PBS** | | **MKO-DEX** | | **Significant by *treatment, **genotype or ***both by ANOVA** | | **Gene ontology** |
| --- | --- | --- | --- | --- | --- | --- | --- | --- | --- | --- |
| **Aconitate hydratase, mitochondrial** | | 0.17± 0.02 | 0.12± 0.02 | | 0.16± 0.04 | | 0.14± 0.03 | | ***Yes** | Mitochondrial |
| **Malate dehydrogenase, mitochondrial** | | 0.14± 0.02 | 0.1± 0.02 | | 0.1± 0.04 | | 0.09± 0.04 | | ***Yes** | Mitochondrial |
| **ATP synthase subunit beta, mitochondrial** | | 0.15± 0.02 | 0.11± 0.02 | | 0.11± 0.04 | | 0.1± 0.04 | | No | Mitochondrial |
| **Creatine kinase S-type, mitochondrial** | | 0.17± 0.03 | 0.13± 0.02 | | 0.15± 0.06 | | 0.13± 0.06 | | No | Mitochondrial |
| **ATP synthase subunit alpha, mitochondrial** | | 0.18± 0.03 | 0.15± 0.05 | | 0.17± 0.05 | | 0.13± 0.06 | | No | Mitochondrial |
| **Isocitrate dehydrogenase [NADP], mitochondrial** | | 0.21± 0.02 | 0.2± 0.03 | | 0.17± 0.02 | | 0.16± 0.03 | | ****Yes** | Mitochondrial |
| **Electron transfer flavoprotein subunit alpha, mitochondrial** | | 0.23± 0.06 | 0.21± 0.07 | | 0.17± 0.06 | | 0.15± 0.03 | | ****Yes** | Mitochondrial |
| **Aspartate aminotransferase, mitochondrial** | | 0.14± 0.02 | 0.11± 0.01 | | 0.12± 0.03 | | 0.12± 0.04 | | No | Mitochondrial |
| **Citrate synthase, mitochondrial** | | 0.15± 0.02 | 0.11± 0.03 | | 0.12± 0.04 | | 0.1± 0.04 | | ***Yes** | Mitochondrial |
| **Fumarate hydratase, mitochondrial** | | 0.15± 0.03 | 0.08± 0.03 | | 0.12± 0.04 | | 0.11± 0.06 | | ***Yes** | Mitochondrial |
| **Cytochrome c, somatic** | | 0.15± 0.04 | 0.14± 0.02 | | 0.12± 0.03 | | 0.16± 0.05 | | No | Mitochondrial |
| **Electron transfer flavoprotein subunit beta** | | 0.19± 0.04 | 0.2± 0.04 | | 0.18± 0.03 | | 0.17± 0.02 | | No | Mitochondrial |
| **Long-chain specific acyl-CoA dehydrogenase, mitochondrial** | | 0.2± 0.07 | 0.19± 0.03 | | 0.19± 0.04 | | 0.19± 0.03 | | No | Mitochondrial |
| **ES1 protein homolog, mitochondrial** | | 0.18± 0.1 | 0.11± 0.04 | | 0.13± 0.07 | | 0.07± 0.07 | | No | Mitochondrial |
| **Medium-chain specific acyl-CoA dehydrogenase, mitochondrial** | | 0.29± 0.02 | 0.21± 0.06 | | 0.22± 0.06 | | 0.2± 0.07 | | No | Mitochondrial |
| **Dihydrolipoyl dehydrogenase, mitochondrial** | | 0.21± 0.02 | 0.14± 0.02 | | 0.12± 0.09 | | 0.05± 0.02 | | *****Yes** | Mitochondrial |
| **Glycogen phosphorylase, muscle form** | | 0.21± 0.02 | 0.12± 0.05 | | 0.17± 0.08 | | 0.12± 0.07 | | ***Yes** | Glucose metabolism |
| **Fructose-bisphosphate aldolase A** | | 0.23± 0.04 | 0.19± 0.04 | | 0.24± 0.07 | | 0.19± 0.08 | | No | Glucose metabolism |
| **Beta-enolase** | | 0.11± 0.04 | 0.06± 0.04 | | 0.1± 0.06 | | 0.06± 0.07 | | No | Glucose metabolism |
| **Glyceraldehyde-3-phosphate dehydrogenase** | | 0.13± 0.03 | 0.08± 0.04 | | 0.12± 0.07 | | 0.08± 0.07 | | No | Glucose metabolism |
| **Phosphoglucomutase-1** | | 0.13± 0.02 | 0.08± 0.04 | | 0.1± 0.05 | | 0.07± 0.06 | | ***Yes** | Glucose metabolism |
| **Pyruvate kinase PKM** | | 0.16± 0.03 | 0.11± 0.04 | | 0.14± 0.06 | | 0.11± 0.07 | | No | Glucose metabolism |
| **Glucose-6-phosphate isomerase** | | 0.13± 0.03 | 0.1± 0.04 | | 0.12± 0.05 | | 0.09± 0.05 | | No | Glucose metabolism |
| **Phosphoglycerate kinase 1** | | 0.09± 0.03 | 0.08± 0.03 | | 0.1± 0.04 | | 0.08± 0.04 | | No | Glucose metabolism |
| **Triosephosphate isomerase** | | 0.11± 0.03 | 0.08± 0.03 | | 0.11± 0.05 | | 0.08± 0.06 | | No | Glucose metabolism |
| **Phosphoglycerate mutase 2** | | 0.11± 0.03 | 0.05± 0.04 | | 0.1± 0.06 | | 0.05± 0.07 | | ***Yes** | Glucose metabolism |
| **L-lactate dehydrogenase A chain** | | 0.12± 0.03 | 0.08± 0.04 | | 0.11± 0.07 | | 0.07± 0.07 | | No | Glucose metabolism |
| **ATP-dependent 6-phosphofructokinase, muscle type** | | 0.36± 0.02 | 0.26± 0.07 | | 0.31± 0.08 | | 0.25± 0.11 | | ***Yes** | Glucose metabolism |
| **Malate dehydrogenase, cytoplasmic** | | 0.16± 0.02 | 0.13± 0.02 | | 0.16± 0.05 | | 0.13± 0.04 | | No | Glucose metabolism |
| **Alpha-enolase** | | 0.11± 0.03 | 0.06± 0.03 | | 0.13± 0.1 | | 0.06± 0.06 | | ***Yes** | Glucose metabolism |
| **Gamma-enolase** | | 0.12± 0.04 | 0.06± 0.03 | | 0.09± 0.06 | | 0.06± 0.06 | | ***Yes** | Glucose metabolism |
| **L-lactate dehydrogenase B chain** | | 0.4± 0.16 | 0.27± 0.04 | | 0.2± 0.08 | | 0.19± 0.04 | | ****Yes** | Glucose metabolism |
| **Creatine kinase M-type** | | 0.13± 0.03 | 0.07± 0.04 | | 0.13± 0.06 | | 0.09± 0.07 | | ***Yes** | Cytoplasmic |
| **Myoglobin** | | 0.14± 0.03 | 0.09± 0.03 | | 0.09± 0.03 | | 0.08± 0.02 | | *****Yes** | Cytoplasmic |
| **Carbonic anhydrase 3** | | 0.14± 0.02 | 0.05± 0.02 | | 0.08± 0.05 | | 0.05± 0.03 | | *****Yes** | Cytoplasmic |
| **Adenylate kinase isoenzyme 1** | | 0.12± 0.02 | 0.1± 0.03 | | 0.12± 0.05 | | 0.09± 0.05 | | No | Cytoplasmic |
| **Sarcoplasmic/endoplasmic reticulum calcium ATPase 1** | | 0.2± 0.01 | 0.17± 0.03 | | 0.18± 0.04 | | 0.16± 0.06 | | No | Cytoplasmic |
| **Parvalbumin alpha** | | 0.1± 0.02 | 0.05± 0.03 | | 0.08± 0.05 | | 0.06± 0.06 | | No | Cytoplasmic |
| **Protein DJ-1** | | 0.11± 0.02 | 0.08± 0.05 | | 0.1± 0.05 | | 0.08± 0.05 | | No | Cytoplasmic |
| **Heat shock cognate 71 kDa protein** | | 0.29± 0.07 | 0.25± 0.06 | | 0.21± 0.06 | | 0.2± 0.05 | | ****Yes** | Cytoplasmic |
| **Aspartate aminotransferase, cytoplasmic** | | 0.17± 0.03 | 0.12± 0.03 | | 0.17± 0.07 | | 0.12± 0.05 | | ***Yes** | Cytoplasmic |
| **Nucleoside diphosphate kinase B** | | 0.17± 0.02 | 0.14± 0.04 | | 0.14± 0.04 | | 0.12± 0.04 | | No | Cytoplasmic |
| **Adenylosuccinate synthetase isozyme 1** | | 0.3± 0.02 | 0.28± 0.06 | | 0.29± 0.08 | | 0.24± 0.08 | | No | Cytoplasmic |
| **Superoxide dismutase [Cu-Zn]** | | 0.17± 0.03 | 0.11± 0.06 | | 0.17± 0.03 | | 0.17± 0.04 | | No | Cytoplasmic |
| **Aldose reductase** | | 0.14± 0.04 | 0.1± 0.05 | | 0.08± 0.04 | | 0.1± 0.08 | | No | Cytoplasmic |
| **Peptidyl-prolyl cis-trans isomerase A** | | 0.23± 0.02 | 0.19± 0.06 | | 0.21± 0.06 | | 0.22± 0.06 | | No | Cytoplasmic |
| **Elongation factor 1-alpha 2** | | 0.2± 0.03 | 0.15± 0.04 | | 0.14± 0.08 | | 0.13± 0.05 | | No | Cytoplasmic |
| **Phosphatidylethanolamine-binding protein 1** | | 0.16± 0.02 | 0.1± 0.04 | | 0.13± 0.02 | | 0.11± 0.03 | | ***Yes** | Cytoplasmic |
| **Sarcalumenin** | | 0.12± 0.06 | 0.05± 0.04 | | 0.05± 0.03 | | 0.04± 0.03 | | No | Cytoplasmic |
| **Peroxiredoxin-1** | | 0.25± 0.04 | 0.3± 0.09 | | 0.28± 0.04 | | 0.33± 0.09 | | No | Cytoplasmic |
| **Glutathione S-transferase Mu 2** | | 0.24± 0.04 | 0.22± 0.06 | | 0.13± 0.03 | | 0.19± 0.03 | | ****Yes** | Cytoplasmic |
| **Alpha-crystallin B chain** | | 0.45± 0.04 | 0.44± 0.08 | | 0.52± 0.06 | | 0.54± 0.06 | | ****Yes** | Cytoplasmic |
| **Myosin light chain 1/3, skeletal muscle isoform** | | 0.14± 0.02 | 0.06± 0.04 | | 0.1± 0.06 | | 0.05± 0.08 | | ***Yes** | Myofibrillar |
| **LIM domain-binding protein 3** | | 0.14± 0.02 | 0.13± 0.01 | | 0.14± 0.02 | | 0.13± 0.04 | | No | Myofibrillar |
| **Troponin C, skeletal muscle** | | 0.24± 0.03 | 0.18± 0.05 | | 0.2± 0.07 | | 0.18± 0.1 | | No | Myofibrillar |
| **Tropomyosin beta chain** | | 0.13± 0.04 | 0.07± 0.04 | | 0.12± 0.06 | | 0.06± 0.08 | | ***Yes** | Myofibrillar |
| **Tropomyosin alpha-1 chain** | | 0.15± 0.03 | 0.07± 0.04 | | 0.13± 0.06 | | 0.08± 0.11 | | ***Yes** | Myofibrillar |

**Fractional synthesis of gastrocnemius muscle proteins sorted by gene ontology.** Glucose metabolism (n=16), mitochondrial (n=16), cytoplasmic (n=20) and myofibrillar proteins (n=5). Values shown are the mean ± S.D. (n≥ 3 per group) for 57 proteins. WT-PBS, wild-type treated with phosphate buffer saline (PBS), WT-DEX, wild-type treated with DEX, MKO-PBS, P85α muscle specific knockout given PBS, and MKO-DEX, P85α muscle specific knockout given DEX. An ANOVA analysis was conducted via InfernoRND proteomic analysis and determined 19, 9 and 3 proteins were affected by *treatment, **genotype or ***both, respectively. A yes or no indicates statistical significance, p ≤0.05.

Supporting information file 1: experiment 1 protein identification and percent coverage information.

Supporting information file 2: experiment 2 protein identification and percent coverage information.

These files contain entry name, accession number, group number, sub group number, number of spectra, number of unique peptides, unique score, percent coverage, coverage map, total intensity, protein molecular weight, database, parent charge, score, sequence, chromatographic peak width, parent mass to charge ratio, delta parent mass and delta parent mass in ppm.

Supporting information file 3: summary table for experiment 1.

Supporting information file 4: summary table for experiment 2.

The summary tables contain the raw data for mass isotopomer distribution kinetic analysis for experiment 1 and 2.

Supporting information file 5: Data filter script for experiment 1

Supporting information file 6: Data filter script for experiment 2

The data filter script contains mass isotopomer distribution analysis protein kinetic analysis processed data that includes peptide output, protein output, parameters and project details for experiment 1 and 2.

Supporting information file 7: Combined data script for experiment 1 and 2 final analysis.

This is the combined data filter for processed protein kinetic data for experiment 1 and 2.

Data can be accessed publicly online via Figshare here: <https://figshare.com/projects/The_role_of_striated_muscle_Pik3r1_in_glucose_and_protein_metabolism_following_chronic_glucocorticoid_exposure/82868>. Project ID is 82868. One can access raw mass spectrometry data for identifications and mass isotopomer distribution analysis protein kinetic data as well.

**Quantitative Real-Time PCR (qPCR)**

Liver tissues were homogenized using TRIzol reagent (Invitrogen, Waltham, MA, 15596018) and RNA was extracted. Reverse transcription was performed as following: 0.5 μg of total RNA, 4 μl of 2.5 mM dNTP and 2 μl of 15 μM random primers (New England Biolabs, Ipswich, MA, S1254S) were mixed at a volume of 16 μl, and incubated at 70°C for 5 min. Then, a 4 μl cocktail containing 25 units of Moloney Murine Leukemia Virus (M-MuLV) Reverse Transcriptase (New England Biolabs, Ipswich, MA, M0253S), 10 units of RNasin Plus (Promega, Madison, WI, N261B) and 2 μl of 10x M-MuLV Reverse Transcriptase Reaction Buffer (New England Biolabs, Ipswich, MA, B0253S) was added, and samples were incubated at 42°C for 1h and then at 95°C for 5 min.  The cDNA was diluted and used for real-time quantitative PCR (qPCR) using the Power Eva qPCR SuperMix Kit (Biochain, Newark, CA, K5057400), following manufacturer’s protocol. The qPCR was performed on the StepOne PCR System (Applied Biosystems, Foster City, CA) and analyzed with the ∆∆-Ct method. Rpl19 gene expression was used for internal normalization.

| **Primer** | **Forward** | **Reverse** |
| --- | --- | --- |
| mRPL19_cDNA | ATGGAGCACATCCACAAGC | TCCTTGGTCTTAGACCTGCG |
| mPik3r1_cDNA | CAAAGCGGAGAACCTATTGC | ATAGCAGCCCTGCTTACTGC |
| mSesn1_cDNA | TATGGCCATGCACAAAGATG | TTCCAAACATGCAGTGGATA |
| mFkbp5_cDNA | TGA GGG CAC CAG TAA CAA TGG | CAA CAT CCC TTT GTA GTG GAC AT |
| mPIK3r1 ChIP | GGG AAG TGG GAT AAA AGG AG | CCT TTC TTT CTC CCG TCT GT |
